# Role of Shear Stress and tPA Concentration in the Fibrinolytic Potential of Thrombi

**DOI:** 10.3390/ijms22042115

**Published:** 2021-02-20

**Authors:** Claire S. Whyte, Hadj Ahmed. Mostefai, Kim M. Baeten, Andrew J. Lucking, David E. Newby, Nuala A. Booth, Nicola J. Mutch

**Affiliations:** 1 Aberdeen Cardiovascular and Diabetes Centre, Institute of Medical Sciences, School of Medicine, Medical Sciences and Nutrition, University of Aberdeen, Foresterhill, Aberdeen AB25 2ZD, Scotland, UK; c.s.whyte@abdn.ac.uk (C.S.W.); mostefaih@yahoo.fr (H.A.M.); kimbaeten1@gmail.com (K.M.B.); n.a.booth@abdn.ac.uk (N.A.B.); 2 Centre for Cardiovascular Science, University of Edinburgh, Edinburgh EH16 4SB, Scotland, UK; Andrew.Lucking@ouh.nhs.uk (A.J.L.); D.E.Newby@ed.ac.uk (D.E.N.)

**Keywords:** plasminogen activator inhibitor-1, fibrinolysis, tissue plasminogen activator, thrombi, shear

## Abstract

The resolution of arterial thrombi is critically dependent on the endogenous fibrinolytic system. Using well-established and complementary whole blood models, we investigated the endogenous fibrinolytic potential of the tissue-type plasminogen activator (tPA) and the intra-thrombus distribution of fibrinolytic proteins, formed ex vivo under shear. tPA was present at physiologically relevant concentrations and fibrinolysis was monitored using an FITC-labelled fibrinogen tracer. Thrombi were formed from anticoagulated blood using a Chandler Loop and from non-anticoagulated blood perfused over specially-prepared porcine aorta strips under low (212 s^−1^) and high shear (1690 s^−1^) conditions in a Badimon Chamber. Plasminogen, tPA and plasminogen activator inhibitor-1 (PAI-1) concentrations were measured by ELISA. The tPA–PAI-1 complex was abundant in Chandler model thrombi serum. In contrast, free tPA was evident in the head of thrombi and correlated with fibrinolytic activity. Badimon thrombi formed under high shear conditions were more resistant to fibrinolysis than those formed at low shear. Plasminogen and tPA concentrations were elevated in thrombi formed at low shear, while PAI-1 concentrations were augmented at high shear rates. In conclusion, tPA primarily localises to the thrombus head in a free and active form. Thrombi formed at high shear incorporate less tPA and plasminogen and increased PAI-1, thereby enhancing resistance to degradation.

## 1. Introduction

Fibrinolysis is responsible for counterbalancing thrombus formation, promoting thrombus dissolution and facilitating wound healing. Plasmin is the key proteolytic enzyme responsible for degradation of fibrin and is formed by the cleavage of the zymogen form, plasminogen, at Arg561-Val562 [1,2]. The two principal physiological plasminogen activators are tissue-type plasminogen activator (tPA) and urokinase-type plasminogen activator (uPA). Under normal conditions, the circulating concentration of tPA is low but local concentrations rise markedly following release from vascular endothelial cells in response to stimuli including shear stress, fibrin deposition, thrombin and bradykinin [3,4,5,6] promoting fibrinolysis and thrombus resolution [7]. The rate at which fibrinolysis proceeds is dependent on several factors and the process is normally tightly regulated by inhibitors of these proteases. The serpin, plasminogen activator inhibitor-1 (PAI-1), is the principal inhibitor of tPA and uPA, rapidly inactivating them by forming a 1:1 complex which is then cleared from the circulation via the hepatic system. Whilst platelets are the primary source of circulating PAI-1 [8,9], releasing and retaining functional PAI-1 on their membrane [10], it is also synthesised by endothelial cells [11], adipocytes [12,13,14], hepatocytes [15] and cardiomyocytes [16] and the concentration of circulating PAI-1 increases in response to several inflammatory cytokines [17,18,19,20,21,22].

The shear conditions in which thrombi form greatly impacts their structure and composition [23,24], both of which are important factors in determining how resistant a given thrombus is to fibrinolytic degradation [25]. Strain tends to align fibres on the surface of the thrombus in the direction of flow, whereas interior fibres have a more random and porous orientation [26,27]. Fibrin networks consisting of thin fibres are more tightly packed [28] and these dense clots are more resistant to fibrinolytic degradation [29]. In addition to the composition of fibrin, thrombi formed under high shear conditions contain high numbers of platelets, while those forming under lower shear conditions are more fibrin- and erythrocyte-rich [23]. Platelet-rich thrombi are more resistant to lysis than erythrocyte-rich thrombi [30], which likely explains the failure of some vessels to recanalize after thrombolytic therapy [31].

We have previously demonstrated a concentration dependent effect of tPA-mediated fibrinolysis in overcoming thrombin activatable fibrinolysis inhibitor (TAFI) inhibition in the Chandler model [32]. In those studies, tPA was added at a relatively high concentration (1 µg/mL) after thrombus formation [32]. In the present study, we aimed to characterise: (i) the distribution of tPA and other important fibrinolytic proteins in thrombi and (ii) the downstream implications in terms of fibrinolysis, when thrombi are formed in the presence of physiological concentrations of tPA under flow conditions equivalent to those encountered in the venous and arterial circulations. We investigated the impact of shear rate on fibrinolysis in thrombi formed from non-anticoagulated blood in an ex vivo model of coronary arterial injury.

## 2. Results

### 2.1. Fibrin Formation and Fibrinolysis Occur Simultaneously during Thrombus Formation

**Figure 1:**
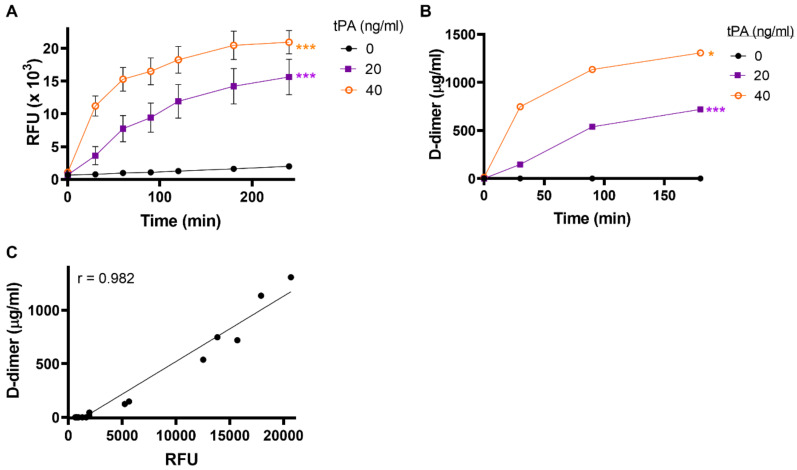
Dose-dependent thrombolysis with endogenous levels of tPA. Whole-blood model thrombi containing FITC-fibrinogen were formed by rotation for 30 min in the presence or absence of 20 or 40 ng/mL added tissue-type plasminogen activator (tPA). (**A**) Fibrinolysis was measured as relative fluorescence release (RFU) over 4 h in the bathing fluid. (**B**) D-dimer levels were measured in the bathing fluid and (**C**) correlated with FITC results (R = 0.96). Data shown are mean ± SEM, *n* ≥ 3. *** *p* < 0.001 vs. no tPA.

The endothelium is the major source of tPA during inflammatory insult to the vasculature [33]. To mimic this situation, we formed Chandler model thrombi under rotation in the presence of exogenous tPA (20 or 40 ng/mL). Incorporation of tPA during thrombus formation resulted in ongoing thrombus formation and dissolution. We characterised the optimal time for thrombus formation to be 30 min, as after this point, substantial fluorescence release was detected in the Chandler loop serum which is indicative of ongoing fibrinolysis (Figure S1). Using this optimised time for thrombus formation we determined that lysis was significantly enhanced upon incorporation of tPA compared to the absence of tPA (Figure 1A, *p* < 0.001). Consistent with this, D-dimer concentrations in the bathing fluid were increased—indicative of ongoing fibrinolysis (Figure 1B)—and strongly correlated with fluorescence release (*r* = 0.98) (Figure 1C).

### 2.2. Incorporated tPA Protects Thrombi against Inhibition by PAI-1

**Figure 2:**
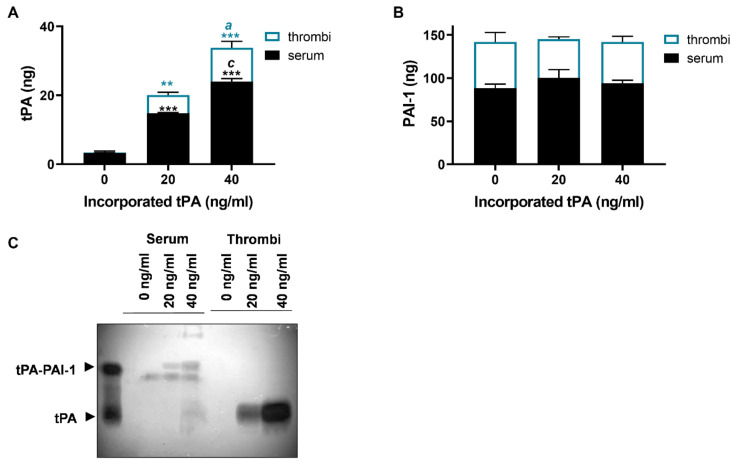
tPA incorporated into thrombi is protected from PAI-1 inhibition. Chandler model thrombi were formed and the levels of (**A**) tPA, and (**B**) plasminogen activator inhibitor-1(PAI-1) in serum and thrombus extracts were measured by ELISA. (**C**) Detection of tPA by zymography showing tPA and tPA–PAI-1 in the serum and thrombus extract after incorporation 0, 20 or 40 ng/mL tPA. Data shown are mean ± SEM (*n* = 4). ** *p* < 0.01, *** *p* < 0.001 vs. no tPA, *^a^ p* < 0.05, *^c^ p* < 0.001.

To establish the degree to which tPA is incorporated into thrombi, we quantified tPA in thrombus extracts of Chandler model thrombi and the residual serum. Negligible quantities of tPA were detected in thrombus extracts and serum (0.05 ± 0.01 and 3.25 ± 0.56 ng, respectively) in the absence of exogenous tPA (Figure 2A). Limited detection of tPA in this circumstance is not surprising, given the lack of endothelium in this system. Inclusion of both 20 and 40 ng/mL tPA during thrombus formation augmented the levels in the thrombus extract (5.28 ± 0.83, *p* < 0.01, and 9.84 ± 1.83 ng, *p* < 0.001, respectively) and serum (14.78 ± 0.20 and 24.0 ± 0.84 ng, respectively, *p* < 0.001 for both) indicating that approximately a quarter was incorporated into the thrombus. The PAI-1 contents in thrombi and serum were unaltered by incorporation of tPA (Figure 2B) and were always in excess of tPA.

The association of PAI-1 and tPA was further examined in the thrombus extract and serum by zymography (Figure 2C). In serum, tPA was in complex with PAI-1 and other inhibitors. In marked contrast, in the thrombus extract, tPA was evident in a free uncomplexed form (Figure 2C), suggesting functional tPA persisted within the microenvironment of the thrombi despite high levels of PAI-1 (Figure 2B).

### 2.3. Fibrinolytic Activity Is Primarily Concentrated in the “Head” of Thrombus

**Figure 3:**
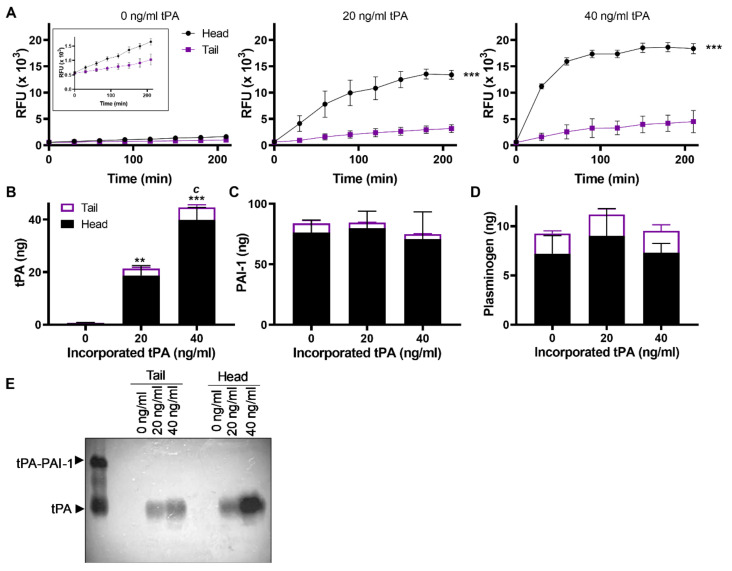
Incorporated tPA increased in the thrombus head. (**A**) Fibrinolysis was measured as fluorescence released in the bathing fluid from heads and tails in the absence or presence of 20 or 40 ng/mL of incorporated tPA. Inset shows the fluorescence released at 0 ng/mL of tPA on a smaller scale. (**B**) tPA, (**C**) PAI-1 and (**D**) plasminogen antigen concentrations were measured by ELISA in extracts of heads and tails. (**E**) Detection of tPA by zymography showing tPA and tPA–PAI-1 in extract of thrombus head and tail sections formed with incorporation of 0, 20 or 40 ng/mL tPA. Data shown are mean ± SEM (*n* = 3). *** *p* < 0.001 vs. no tPA.

Thrombi formed under flow in the Chandler loop show similar morphology to human thrombi formed in vivo [23], comprising of a dense head, rich with platelet, erythrocytes, leucocytes and fibrin, and with a tail region comprised mainly of fibrin [23]. We examined the distribution of tPA in the thrombi. Lysis was increased in the head of the thrombus, both in the absence and presence of exogenous tPA (Figure 3A, *p* < 0.001). Increasing concentrations of endogenous tPA augmented the levels within thrombi which were localised primarily within the head (Figure 3B). In contrast, PAI-1 levels remained constant despite differences in tPA concentrations within the thrombus, suggesting there was no substantial increase in tPA–PAI-1 complex levels (Figure 3C). Similarly, plasminogen concentrated in the thrombus head and remained constant (Figure 3D). Free tPA was detected in the thrombus head by zymography, suggesting it was protected from inhibition by PAI-1 (Figure 3E).

### 2.4. Shear Rate Influences the Fibrinolytic Protein Content of Thrombi and Impedes Fibrinolysis at High Shear

**Figure 4:**
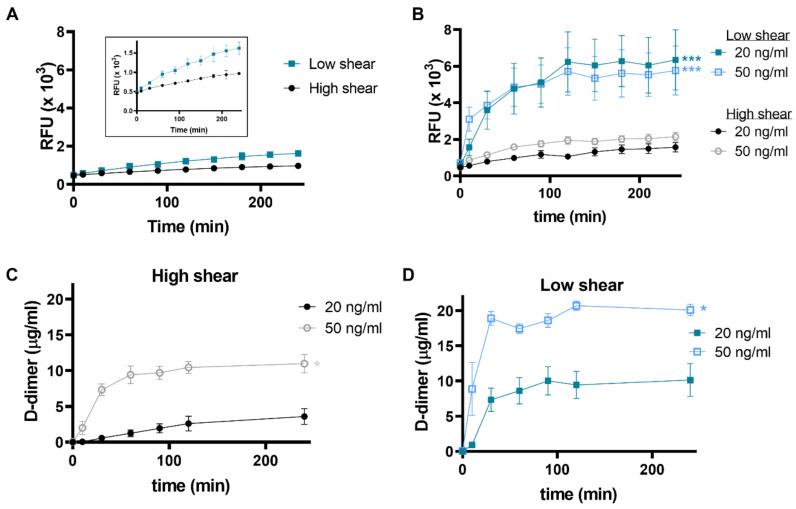
Fibrinolysis under high and low shear conditions. Whole blood thrombi were formed in Badimon chambers on a thrombogenic surface under low (212 s^−1^) and high (1690 s^−1^) shear rates. Fibrinolysis was measured as fluorescence release in the bathing fluid over time. Thrombi formed in the (**A**) absence or (**B**) with the incorporation of tPA at 20 or 40 ng/mL (**B**). D-dimer levels were measured at (**C**) high and (**D**) low shear rates. Data shown are mean ± SEM (*n* = 7).

To allow us to investigate the impact of flow conditions on fibrinolytic activity, we next utilised a model that permitted us to study thrombus formation in non-anticoagulated whole blood under well-defined low and high shear rates. Fibrinolysis in the absence of exogenous tPA proceeded more efficiently at low shear compared to high shear (Figure 4A). Inclusion of tPA (20 and 50 ng/mL) to thrombi formed at high shear had little impact on the rate of lysis (Figure 4B). In contrast, lysis proceeded faster (4.8-fold increase, *p* < 0.001) in thrombi formed at low shear (Figure 4B). Consistent with the enhanced fluorescence release, D-dimer concentrations rose more quickly and to a higher level at low shear for both tPA concentrations, indicating a faster rate of clot dissolution. Interestingly, despite no detectable differences in the rate of fibrinolysis after incorporation of the different tPA concentrations, a concentration dependent effect on D-dimer was observed under both low and high shear conditions (Figure 4C,D).

**Figure 5:**
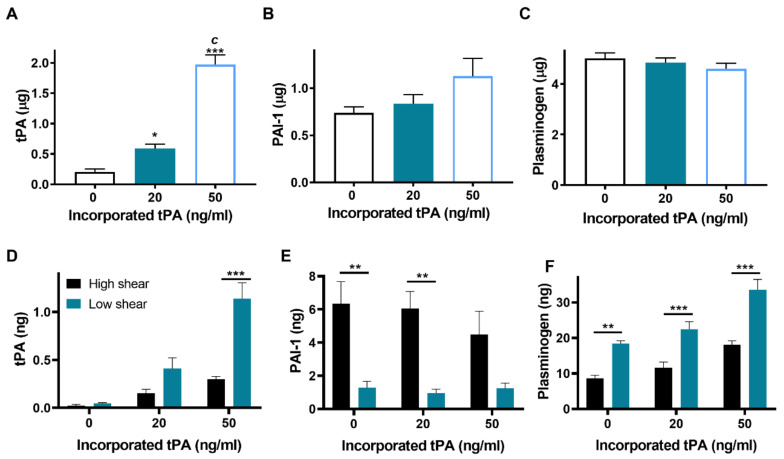
Thrombi formed at low shear have higher levels of tPA and plasminogen. Whole blood thrombi were formed in Badimon chambers on a thrombogenic surface under low (212 s^−1^) and high (1690 s^−1^) shear rates. Levels of tPA, PAI-1 and plasminogen antigen were measured by ELISA in the effluent (**A**–**C**, respectively), * *p* < 0.05, *** *p* < 0.001 vs. no tPA, c *p* < 0.001 vs. 20 ng/mL tPA and the thrombus bathing fluid (**D**–**F**, respectively), ** *p* < 0.01, *** *p* < 0.001 vs high shear. Data shown are mean ± SEM (*n* = 7).

The concentrations of tPA, PAI-1 and plasminogen were quantified in the bathing fluid and the Badimon chamber effluent. A concentration dependent increase in tPA was evident in the chamber effluent (Figure 5A). Basal levels were 0.2 ± 0.05 µg, increasing to 0.59 ± 0.07 and 1.97 ± 0.16 µg tPA after addition of 20 and 50 ng/mL tPA, respectively (Figure 5A). Similar PAI-1 and plasminogen concentrations were detected in the effluent at all tPA concentrations (Figure 5B,C).

Negligible tPA was detected in the thrombus bathing fluid in the absence of exogenous protein (high shear 0.02 ± 0.03 ng, low shear 0.05 ± 0.01 ng) (Figure 5D). The addition of 20 ng/mL tPA increased levels in the bathing fluid by around 6.6- and 8.5-fold at low and high shear, respectively (Figure 5D). At low shear, tPA increased 2.8-fold in the bathing fluid in the presence of exogenous tPA, whereas at high shear this effect was less pronounced. These data suggest that incorporation of tPA into the thrombi is augmented under low shear conditions. PAI-1 concentration in the bathing fluid was augmented at high shear compared to low shear (Figure 5E). Inclusion of tPA at 20 or 50 ng/mL did not alter the PAI-1 concentration in the bathing fluid at low or high shear but was unaltered by the addition of tPA (Figure 5E). Interestingly, plasminogen levels in the bathing fluid were elevated with increasing tPA incorporation and at low shear (Figure 5F). Together these data suggest that high shear attenuates fibrinolysis by limiting the incorporation of tPA and plasminogen into thrombus, while increasing PAI-1 content.

## 3. Discussion

The impacts of flow on platelet deposition, thrombus formation and fibrin structure are well established. The relationship between flow and fibrinolysis is less clear due to the complexity and limited availability of models. Our laboratories have been instrumental in investigating fibrinolysis of thrombi formed in the presence of physiologically-relevant levels of tPA and under a variety of continuous flow conditions [7,34,35,36,37,38]. Here, we utilised two complementary ex vivo models to investigate fibrinolysis with endogenous levels of tPA. Accumulation of tPA and plasminogen was observed in the head of Chandler model thrombi, directly aligning with localisation of fibrinolytic activity. Within the microenvironment of thrombi, tPA appeared protected from inhibition by PAI-1, whereas, in serum it was found in complex with its inhibitor. The striking abundance of tPA in the head of the thrombus was surprising considering this region is largely comprised of platelets and leukocytes, while the thrombus tail is fibrin-rich nature [23]. Previously, we demonstrated that endogenous thrombus lysis is largely mediated by uPA in the head of thrombi, with a modest contribution from tPA [39,40]. The origin of this uPA activity is polymorphonuclear cells that accrue in this region under flow [39,40]. Here, tPA was included during thrombus formation, at physiologically-relevant levels.

Using an ex vivo model of vascular injury, we demonstrated—for the first time—the direct impact of shear conditions on the localisation of fibrinolytic proteins and the downstream impact on lysis of thrombi formed from native whole blood, again in the presence of physiologically-relevant levels of tPA.

Our previous work has shown that Chandler model thrombi accurately mimic human arterial thrombi, consisting of platelet-rich head and fibrin-rich tail [23]. A recent study examining arterial thrombi obtained after thrombectomy by scanning electron microscopy, indicated that platelets accounted for 31% of the thrombus volume, whilst fibrin accounted for 41% [41]. Conversely, venous thrombi are primarily composed of red blood cells and fibrin, with platelets accounting for only 0.4% [41]. Platelets are the major pool of circulating PAI-1 [8,9] and we have recently shown that the stimulated platelet membrane retains functional PAI-1 [10]. Conversely, we have shown that platelets bind plasminogen via both fibrin-dependent and independent mechanisms [38,42]. We identified the presence of the plasminogen receptor, Plg-R_KT_, on the surface of platelets, which is augmented following stimulation and acts as a binding partner for both plasminogen and tPA [42]. These data imply that platelets function in both profibrinolytic and antifibrinolytic pathways within the microenvironment of the thrombus. The balance of these functions may relate to their positioning relative to the vessel wall, activation status and shear stress.

Thrombi formed ex vivo in the Badimon Chamber model form sequentially, at low shear (approximately 212 s^−1^) and subsequently high shear (approximately 1690 s^−1^), permitting direct comparison of thrombi formed from the same native blood sample. In addition to allowing the effect of different shear conditions to be examined, this model has several strengths, including that thrombus formation occurs as native blood and flows over a pathophysiologically relevant thrombogenic surface [7]. Thrombi formed at high shear were more resistant to fibrinolysis than those formed at low shear, likely due to higher PAI-1 and lower tPA and plasminogen levels within these thrombi. The high PAI-1 levels in the thrombi formed under high shear most likely reflect increased platelet deposition, consistent with previous observation of high platelets in arterial thrombi [23,34,43,44,45].

D-dimer is commonly used as a clinical marker of increased fibrinolytic activity. An exceptionally strong correlation between fluorescence release and the D-dimer levels was observed during lysis of thrombi formed in a Chandler Loop. During lysis of the Badimon Chamber thrombi, there was a difference in fluorescence release at high and low shear rates which was reflected in the D-dimer concentrations. However, the sensitivity of the FITC-fibrinogen tracer was not sufficient to detect changes in lysis at different tPA concentrations. This most likely reflects the variations in these flow models, with the Chandler Loop being a closed system, and the Badimon Chamber utilising a continuous flow of whole blood and replenishment of plasma factors. In future studies utilising continuous flow, it may be necessary to increase the ratio of the tracer relative to plasma fibrinogen to augment the sensitivity of the system.

Thrombi formed in the Badimon chamber are comprised of platelet aggregates on the thrombogenic surface and an associated fibrin layer [46], with increased fibrin deposition at low shear [7]. Application of shear forces or mechanical stress is associated with alignment of fibrin fibres in the direction of flow [26,27,47,48], which can alter lateral aggregation and protofibril extension [49]. As a result, thrombi formed in different locale within the body have varying degrees of fibre alignment [50]. Even within a thrombus, fibrin fibre alignment is not uniform, with those fibres on the outer edge of thrombi being—exposed to shear stress—aligning with flow, whereas interior fibres reveal a random orientation [27]. Shear impacts both on the structure of fibrin and on its ability to function as a cofactor in tPA-mediated plasminogen activation [28]. The amplified resistance of thrombi formed under high shear conditions in the Badimon Chamber may reflect changes in the structure of the fibrin, with a subsequent deacceleration of plasminogen activation. Combined with high PAI-1 levels, this appears to contribute to an enhanced thrombus stability. A recent microfluidic model, which was developed to study the impact of thrombolytic drugs in vitro, demonstrated that thrombi formed at high shear are refractory to fibrinolytic degradation at pharmacological concentrations of tPA [51].

Elegant in vivo models of thrombus formation reveal that thrombi have a hierarchical structure, consisting of a dense core of highly activated platelets surrounded by a shell of less activated platelets [52,53]. This concentrated platelet core impedes solute transport from plasma implying that the contents of this area are less susceptible to convective removal [54]. This suggests the intrathrombus content of fibrinolytic proteins may be crucial in governing thrombus resolution. Here, we observed that tPA within thrombi was protected from inhibition despite high levels of its principal inhibitor, PAI-1. Binding of tPA and the downstream enzyme plasmin to a surface, either cellular or fibrin, confers protection against inhibition by PAI-1 or α_2_ antiplasmin, respectively. In line with these observations, we also determined the presence of free tPA in thrombi using zymography, whereas tPA in the serum was in complex with PAI-1. These data indicate that despite favourable kinetics, it is the local milieu that determines the fate of an interaction.

Our laboratory was the first to define a model in which to study fibrinolysis under flow [38]. Using this parallel plate assay combined with high resolution confocal microscopy, we were able to visualise the delicate balance between fibrin formation and dissolution in these thrombi [38]. These observations are reflected in the Chandler model thrombus system, as enhanced fluorescence release, indicative of lysis, was observed in the serum with increasing formation time of the thrombi. This highlights the dynamic nature of thrombus formation with ongoing fibrinolysis, alongside coagulation, with the degree of fibrin deposition dictated by the dominant system. In our previous study, we observed a pool of plasminogen within the core of thrombus that was directly associated with the platelet membrane [38]. These results are emulated here, with the prevalence of plasminogen and tPA within the head of Chandler thrombi, which is rich in platelets and leukocytes. Whilst it was previously believed that fibrin was the main surface driving plasminogen activation, a plethora of data now underscore the importance of cellular surfaces in the regulation of fibrinolysis [42,55,56,57,58].

The data presented here clearly demonstrate that fibrinolytic proteins are not uniformly distributed throughout thrombi and are also impacted by shear stress. Flow based assays recapitulate the hemodynamic conditions required for platelet deposition and fibrin formation, subjecting the thrombus to the convective forces that influence local protein concentrations [59]. One limitation of the current study is that fibrinolysis is not directly studied under flow. Study under flow conditions is complicated by the convective removal and dilution of proteins of interest meaning that there can be a reduction in sensitivity compared to traditional static assays. This has confounded the ability to develop models in which to study fibrinolysis. Recent advances in microscopy and biorheological approaches have pushed the boundaries of fibrinolytic research, permitting visualisation of fibrinolysis in flow models [38,51,59] and in vivo [57,58]. Microfluidic models incorporating supraphysiological levels of tPA have utility in testing thrombolytic drugs [51] and can be applied to conditions where hyperfibrinolysis is observed, such as trauma [59]. This group has previously published findings on the importance of endothelial derived endogenous tPA in suppressing thrombus propagation [7]. Interestingly, endogenous levels of tPA facilitate fibrinolytic activity in vivo in a process that is mechanistically coupled to clot retraction [57].

In our previous study, we demonstrated local endogenous release of tPA induced by bradykinin alone was not enough to produce a system increase in fibrinolytic activity [7]. Stimulation of tPA release with the beta-adrenergic agonists, isoproterenol, also does not alter systemic tPA concentrations [60]. Similarly, in an elegant in vivo model of local ischaemia in pig hearts, a significant increase in local venous tPA activity was observed, yet arterial tPA activity remained unchanged [61]. However, when bradykinin infusion was combined with inhibition of angiotensin converting enzyme (ACE) to limit metabolism of the vasodilator, there was a systemic spill over of tPA, as measured in the non-infused arm [7]. In areas of vessel occlusion, it is expected that bradykinin levels would be higher than those obtained in healthy volunteers due to reduced dilutional effects [7]. Indeed, in patients with established heart disease on long term ACE inhibitor therapy, bradykinin induced plasma tPA activity similar to those seen with systemic thrombolytic therapy [62]. Hrafnkelsdottir et al. demonstrated that the release from the endothelium is the important determinant in local levels of active tPA in organs [63]. Localised pulmonary inter-arterial thrombolysis with streptokinase was shown to be more effective than systemic thrombolysis in reducing morbidity and mortality in patients with high risk pulmonary embolism [64]. These exciting data reveal the importance of localised fibrinolysis in dictating thrombus propagation and persistence in vivo and warrant further investigation.

Here, we illustrate the crucial impact of shear stress on the accumulation and activity of fibrinolytic proteins in thrombi and how this dictates their susceptibility to endogenous fibrinolysis. It is clear that, despite the relatively low concentrations of tPA compared to PAI-1, the activity of this enzyme is preserved within the microenvironment of the thrombus due to surface-mediated interactions. Thrombi formed under high shear conditions are significantly more resistant to fibrinolysis, with higher PAI-1 content and lower tPA and plasminogen concentrations. Fibrin structure may also play a role in this context in terms of dampening the fibrinolytic response. These data indicate that local levels of endogenous tPA released from the endothelium and the shear force applied to a forming thrombus in the vasculature will likely have a powerful influence on its susceptibility to degradation.

## 4. Methods

### 4.1. Subjects and Blood Collection

All blood samples were obtained after approval of local research ethics committees, in accordance with the Declaration of Helsinki and after obtaining written consent.

For Chandler model thrombus studies, citrated whole blood was collected from consented healthy subjects in 0.1 volume of 0.13 M trisodium citrate.

For the Badimon studies, healthy non-smokers were enrolled. Exclusion criteria were bleeding diathesis, the use of regular medication or any clinically significant illness. None of the subjects received vasoactive medication during the study and all abstained from alcohol for 24 h and from food and caffeine-containing drinks for at least six hours before each study. Studies were carried out with subjects lying supine in a quiet, temperature-controlled room maintained between 22 and 25 °C [46,65].

Based on previous studies in which the inflammatory mediator bradykinin was administered to healthy volunteers via intrabrachial infusion to induce endogenous tPA release from the endothelium, [7] thrombi were formed in the presence of tPA (0, 20 and 40 ng/mL Chandler Loop studies and 0, 20 and 50 ng/mL, Badimon Chamber studies).

### 4.2. Chandler Model Thrombi

Thrombi were formed under flow using Chandler loop method, as previously described [32], with the exception that tPA was incorporated during thrombus formation. Briefly, citrated whole blood (900 µL) with the addition of FITC-labelled fibrinogen (43.5 μg/mL Kabi fibrinogen, labelled in house) ± 20 or 40 ng/mL tPA (Genentech) was recalcified with 10.9 mM CaCl_2_ to give a final volume of 1.15 mL. Thrombi were formed under rotation at 30 rpm for 30, 60 or 90 min, after which thrombi were removed from the tubing and the remaining serum was stored for future analysis or fluorescence measurements. Formed thrombi were washed in 0.9% (*w/v*) saline, before being placed in phosphate buffered saline (10 mM phosphate, 150 mM NaCl, pH = 7.4) bathing fluid at 37 °C. Samples were taken every 30 min for 4 h and diluted 1/50 in bathing buffer. Fluorescence release was quantified in the bathing fluid (500 µL) and in serum (1 mL) using a cytofluor multiwell plate reader (series 4000; Applied Biosystems, Framingham, MA, USA) with an excitation wavelength of 485 nm and emission wavelength of 530 nm.

### 4.3. Badimon Chamber Thrombus Formation

Thrombi were formed under defined shear rates using the Badimon Chamber [46]. In brief, blood from an antecubital vein was drawn by a pump through two cylindrical perfusion chambers arranged in series and maintained at 37 °C in a water bath. Strips of porcine aorta (Pel-Freez Biologicals, Rogers, AR, USA), from which the intima and a thin layer of media had been removed, acted as the thrombogenic substrate. FITC-labelled fibrinogen and tPA (0, 20 and 50 ng/mL) were administrated via a calibrated syringe-driver into the extracorporeal circuit. Flow conditions within the first chamber simulate those with a normal coronary artery (low shear rate, approximately 212 s^−1^), whilst those in the second chamber simulate flow conditions within a mild to moderately stenosed coronary artery (high shear rate, approximately 1690 s^−1^). Each study lasted for 5 min, during which blood flow was maintained at a constant rate of 10 mL/min using a peristaltic pump (Masterflex model 7013, Cole-Palmer Instruments, St. Neots, UK) positioned distally to the chambers. The effluent (50.5 mL) was collected into 0.13 M trisodium citrate and kept on ice until platelet free plasma was collected by centrifugation at 2000× *g* for 30 min at 4 °C. Following perfusion of blood, the chambers were flushed with 0.9% saline for 1 min under the same rheological conditions. The porcine strips, with thrombi attached, were subsequently added to the bathing fluid (0.5 mL) and fibrinolysis quantified by fluorescence release.

### 4.4. Enzyme-Linked Immunosorbant Assays

ELISAs for tPA, PAI-1 and plasminogen were performed, as previously described [66]. The fibrin degradation product, D-dimer, was measured using a commercially available kit, Asserachrom D-DI ELISA kit (Diagnostica Stago Inc., Theale, UK). Antigens were quantified in serum from Chandler model thrombi, Badimon chamber effluent and bathing fluid from both models. Chandler model thrombi were analysed as whole thrombi, or head and tail sections extracted in 500 (whole thrombi or head section) and 250 µl (tail section) extraction buffer (50 mM phosphate at pH = 7.0, 0.2 M ε-amino-*N*-caproic acid, 1 M NaCl and 0.01% Tween 20), respectively.

### 4.5. SDS-PAGE Zymography

Model thrombi extracts and serum were analysed for tPA activity by zymography, as previously described [67]. Briefly, proteins were separated on 10% SDS-PAGE gels. The gels were washed in 2.5% (*v/v*) aqueous Triton X-100 and rinsed with distilled water. The gel was then placed on a fibrin/agarose (0.2% Kabi fibrinogen, 0.06 NIH U/mL, 0.8% agarose type 1; Sigma-Aldrich, Darmstadt, Germany) detector plate and incubated at 37 °C. After 16 h the gel was removed, and the detector plate was left to develop for 24–48 h before being photographed.

### 4.6. Statistical Analysis

Rates of lysis (FU/min) and D-dimer release (µg/mL/min) for thrombi formed in both the models were determined by best fit of the slope to a centred second-order polynomial quadratic in GraphPad Prism 8.4 and used to calculate fold differences in lysis and D-dimer release. Antigen levels were calculated as ng based on the concentrations obtained by ELISA and the corresponding total volume of each fraction. Statistical significance was determined by a one-way analysis of variance with a Tukey’s multiple comparison post-hoc test.

## Data Availability

The data presented in this study are available on request from the corresponding author.

## Supplementary Materials

The following are available online at https://www.mdpi.com/1422-0067/22/4/2115/s1, Figure S1: Chandler model thrombi formed at different rotation times.
