# Supplementary material for: Role of Shear Stress and tPA Concentration in the Fibrinolytic Potential of Thrombi
Source: Int J Mol Sci. 2021 Feb 20;22(4):2115. doi: 10.3390/ijms22042115 (PMC7924342; doi:10.3390/ijms22042115)
Supplement: Supplementary file 1 [file ijms-22-02115-s001.pdf]

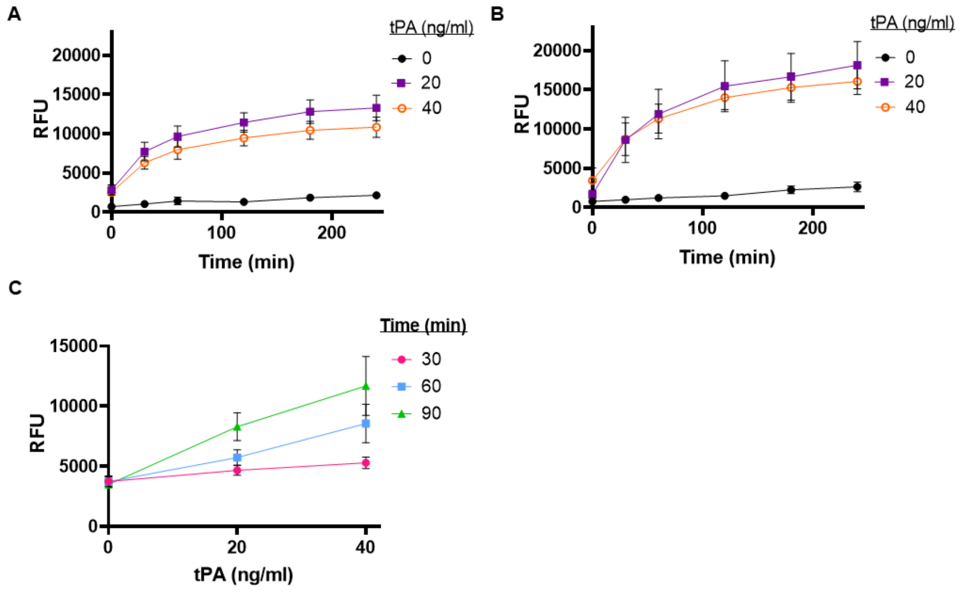

Supplementary Figure 1 – Chandler model thrombi formed at different rotation times. Whole-blood model thrombi containing FITC-fibrinogen were formed by rotation for (A) 90 or (B) 60 min in the presences or absences of 20 ng/ml or 40 ng/ml added tPA (C) Serum collected after formation of thrombi under rotation for 90, 60 or 30. Fibrinolysis was measured as fluorescence release (RFU). Data shown are mean ± SEM (n= 7).
